# Interlayer-Spacing-Modification of MoS_2_ via Inserted PANI with Fast Kinetics for Highly Reversible Aqueous Zinc-Ion Batteries

**DOI:** 10.3390/mi16070754

**Published:** 2025-06-26

**Authors:** Shuang Fan, Yangyang Gong, Suliang Chen, Yingmeng Zhang

**Affiliations:** 1 School of Sino-German Intelligent Manufacturing, Shenzhen City Polytechnic, Shenzhen 518100, China; fanshuangfs0824@126.com; 2 Sunwoda Mobility Energy Technology Co., Ltd., Shenzhen 518132, China; gyy123456yy123456@163.com; 3 Yangtze Delta Region Institute (Huzhou), University of Electronic Science and Technology of China, Huzhou 313000, China

**Keywords:** MoS_2_, PANI, interplanar spacing, aqueous zinc-ion batteries

## Abstract

Layered transition metal dichalcogenides (TMDs) have gained considerable attention as promising cathodes for aqueous zinc-ion batteries (AZIBs) because of their tunable interlayer architecture and rich active sites for Zn^2+^ storage. However, unmodified TMDs face significant challenges, including limited redox activity, sluggish kinetics, and insufficient structural stability during cycling. These limitations are primarily attributed to their narrow interlayer spacing, strong electrostatic interactions, the large ionic hydration radius, and their high binding energy of Zn^2+^ ions. To address these restrictions, an in situ organic polyaniline (PANI) intercalation strategy is proposed to construct molybdenum disulfide (MoS_2_)-based cathodes with extended layer spacing, thereby improving the zinc storage capabilities. The intercalation of PANI effectively enhances interplanar spacing of MoS_2_ from 0.63 nm to 0.98 nm, significantly facilitating rapid Zn^2+^ diffusion. Additionally, the π-conjugated electron structure introduced by PANI effectively shields the electrostatic interaction between Zn^2+^ ions and the MoS_2_ host, thereby promoting Zn^2+^ diffusion kinetics. Furthermore, PANI also serves as a structural stabilizer, maintaining the integrity of the MoS_2_ layers during Zn-ion insertion/extraction processes. Furthermore, the conductive conjugated PANI boosts the ionic and electronic conductivity of the electrodes. As expected, the PANI–MoS_2_ electrodes exhibit exceptional electrochemical performance, delivering a high specific capacity of 150.1 mA h g^−1^ at 0.1 A g^−1^ and retaining 113.3 mA h g^−1^ at 1 A g^−1^, with high capacity retention of 81.2% after 500 cycles. Ex situ characterization techniques confirm the efficient and reversible intercalation/deintercalation of Zn^2+^ ions within the PANI–MoS_2_ layers. This work supplies a rational interlayer engineering strategy to optimize the electrochemical performance of MoS_2_-based electrodes. By addressing the structural and kinetic limitations of TMDs, this approach offers new insights into the development of high-performance AZIBs for energy storage applications.

## 1. Introduction

In recent years, conventional lithium-ion batteries (LIBs) have garnered significant attention as a potential solution to the global energy challenge [1,2]. Nonetheless, several critical issues have emerged, including the harsh preparation conditions, the limited availability of lithium resources, the flammability and explosiveness of organic electrolytes, as well as the inevitable performance degradation under extreme conditions, such as very low or high temperatures, and severe deformation [3,4,5]. These challenges have seriously hindered the further development of practical and large-scale applications of LIBs.

Rechargeable aqueous solution batteries have emerged as an optimal strategy for large-scale energy storage applications. They offer several advantages over conventional organic electrolytes, including higher ionic conductivity (290 mS cm^−1^ vs. 1–10 mS cm^−1^), enhanced safety, low cost, and environmentally friendly production processes [6,7,8]. These benefits address the flammability risks and production challenges associated with conventional systems while improving power characteristics, making them a sustainable alternative for renewable energy integration [9,10].

Among various aqueous battery systems, aqueous zinc-ion batteries (AZIBs) are considered ideal candidates for next-generation energy storage systems. They possess excellent theoretical capacity (5851 mAh cm^−3^ and 820 mAh g^−1^), favorable redox potential (−0.76 V vs. SHE), and significant environmental stability in aqueous electrolytes [11,12,13]. In addition, AZIBs simultaneously offer inherent advantages in terms of ease of processing, safety features, and economic viability (≈$2 kg^−1^), leveraging the Earth’s abundant zinc resources (≈75 ppm, 300 times more than lithium) [14,15]. However, the widespread commercialization of AZIBs is contingent upon the development of advanced cathode materials with superior charge storage capabilities and accelerated reaction kinetics as well as robust zinc electrodes with efficient deposition/dissolution cycles while maintaining near-unity voltammetric efficiency [16].

In recent years, layered materials, including transition metal dichalcogenides (TMDs) [17,18], layered polyanionic compounds (VOPO_4_) [19,20], and layered metal oxides (Mn, V, Mo-based, etc.) [21,22,23,24,25,26], have been extensively investigated for their potential application as cathode materials in AZIBs. These materials are favored for their rich chemical surfaces and adjustable interlayer spacing. Among these, molybdenum disulfide (MoS_2_), as one of the typical representative materials of TMDs, with Mo atoms sandwiched between S atomic layers, is particularly promising due to its higher operating potential and wider output voltage, which translates to higher energy density [27,28]. However, the narrow interlayer spacing of MoS_2_ (0.63 nm) in comparison to the hydrated Zn(H_2_O)_6_^2+^ ion (molecule size of 0.55 nm) and its hydrophobic nature result in a large Zn-ion diffusion barrier, limiting its specific capacity [16,29].

To address these challenges, researchers have focused on multidimensional synergistic modulation strategies, which mainly include (i) novel topological morphology engineering through crystal structure reconstruction [30]; (ii) interlayer spacing modulation to optimize ion-transport channels [31]; (iii) controllable defect construction [32]; (iv) metal phase transition engineering converting the 2H phase into the highly conductive 1T phase [33]; and (v) heterostructure engineering to enhance the overall conductivity [34]. Among these, the interlayer expansion strategy shows significant advantages, which not only reduces the ionic diffusion resistance but also triggers the phase transition from the 2H phase to the highly conductive 1T phase. For example, Liang et al. developed an O–MoS_2_ cathode by incorporating oxygen, which expanded the interlayer spacing (from 0.62 nm to 0.95 nm) and enhanced hydrophilicity, resulting in superior rate capability and a tenfold increase in capacity to 232 mAh g^−1^ [32]. Particularly, Li and Liu et al. reported a MoS_2_/graphene cathode with sandwich-like heterostructures through the insertion of graphene via an electrostatic self-assembly strategy, achieving an expanded layer spacing of 1.16 nm [31]. The reduced graphene oxide coupled with the 1T-rich MoS_2_ phase resulted in better ionic/electronic conductivity and hydrophilicity for the MoS_2_/graphene cathode. Therefore, this composite exhibited a high specific capacity of 285.4 mAh g^−1^ at 0.05 A g^−1^ and excellent cycling stability (approximately 88.2% capacity retention after 1800 cycles).

Recent advancements in advanced hybrid MoS_2_-based cathode design have also explored integrating flexible organic surfactants (e.g., cetyltrimethylammonium bromide (CTAB) and tetramethylammonium (TMA)) [35,36] and conductive polymers (e.g., poly (3,4-ethylenedioxythiophene) (PEDOT)) [37] into the interlayer of the MoS_2_ host to enhance Zn^2^^+^ storage performance. Expectedly, these modifications significantly improve ionic-transport dynamics and enable remarkable high-rate capabilities; the synthesis of PEDOT-intercalated MoS_2_ composites remains constrained by multistep complex processes. Similarly, polyaniline (PANI) intercalation has been investigated as a strategy to expand the MoS_2_ interlayer spacing and improve conductivity for these multivalent ions. Huang et al. (2021) engineered a MoS_2_/PANI hybrid anode achieving 106.5 mAh g^−^^1^ at 1 A g^−^^1^, with 86% capacity retention [38]; Wang’s group (2015) constructed MoS_2_/polyaniline nanocomposites for supercapacitor electrodes, with 390 F g^−^^1^ initial capacitance and 86% cyclic durability over 1000 cycles [39]; Jiang et al. (2017) designed MoS_2_/PANI heterostructured anodes exhibiting exceptional rate capability (885 mAh g^−^^1^ for Li^+^ storage at 4 A g^−^^1^) and 734 mAh g^−^^1^ for Na^+^ storage at 20 mA g^−^^1^ [40]. Expectedly, these modifications significantly improve ionic-transport dynamics and enable remarkable high-rate capabilities. However, the synthesis of both PEDOT- and PANI-intercalated MoS_2_ composites remains constrained by multistep complex processes. These syntheses involve the exfoliation of bulk MoS_2_ to nanosheets, requiring dangerous n-butylithium (n-BuLi) as an ion intercalation aid or flammable surfactants like oleic acid. Therefore, engineering the interlayer structure of MoS_2_ to achieve tunable interlayer separation without compromising mechanical integrity remains a key obstacle to optimizing Zn^2^^+^ storage performance.

In this study, a polyaniline (PANI)-intercalated MoS_2_ composite (PANI–MoS_2_) was synthesized using a combination of guest intercalation and an in situ polymerization approach. Initially, PANI was intercalated into MoOx (PANI–MoOx) via a solvothermal reaction, followed by hydrothermal sulfurization using thiourea as the sulfur precursor to convert PANI–MoOx into PANI–MoS_2_. The intercalation of the conductive polymer markedly expanded the interlamellar spacing of MoS_2_ from 0.62 nm to 0.98 nm, as confirmed by structural characterization. The integration of PANI not only improves the diffusion kinetics of Zn^2^^+^ by broadening the ion-transport pathway but also stabilizes the laminar framework through its π-conjugated backbone, which acts as a structural backbone and mitigates the strong electrostatic repulsion between zinc ions and the MoS_2_ host through charge dispersion. These synergistic effects endowed the PANI–MoS_2_ electrode with exceptional rate performance (150.1 mAh g^−^^1^ at 0.1 A g^−^^1^ and 113.3 mAh g^−^^1^ at 1 A g^−^^1^) and long-term cyclability in AZIBs, retaining 81.2% capacity over 500 cycles at 1 A g^−^^1^. Electrochemical kinetics analyses, including cyclic voltammetry (CV) tests and galvanostatic intermittent titration technique (GITT), revealed dominant pseudocapacitive behavior, lower migration energy barriers, and accelerated Zn^2^^+^ diffusion coefficients. Furthermore, ex situ XRD and high-resolution TEM (HRTEM) demonstrated highly reversible structural evolution and minimal lattice distortion during cycling, corroborating the stability of the composite. This work highlights the rational pre-intercalation of conducting organics into TMDs as a viable strategy for engineering high-performance AZIBs cathodes, providing valuable insights into the design of robust interlayer structures for multivalent ion storage systems.

## 2. Materials and Methods

### 2.1. Material

All chemicals, including Aniline (99.5%, Aladdin, Shanghai, China), (NH_4_)_6_Mo_7_O_24_•4H_2_O (99.9%, Aladdin, Shanghai, China), HCl (37%, Aladdin, Shanghai, China), (NH_4_)_2_S_2_O_8_ (98%, Aladdin, Shanghai, China), ethanol (95%, Aladdin, Shanghai, China), thiourea (99%, Aladdin, Shanghai, China), were used as received.

### 2.2. Preparation of PANI–MoOx Nanocomposite

The PANI–MoOx composite was prepared by a solvothermal method. A mixture of 40 mL of DI water, 3.34 g of aniline, and 2 mmol (NH_4_)_6_Mo_7_O_24_•4H_2_O was supplemented with an aqueous HCl (1 M) solution until a white precipitate was obtained (pH 4–5). Then, 2.5 mmol of (NH_4_)_2_S_2_O_8_ in 10 mL of water was added into the above mixture solution, followed by stirring at 50 °C for a duration of 2 h. After continuously stirring for 30 min, the PANI–MoOx composites were obtained via an in situ polymerization method, washed with ethanol and deionized water several times, and then followed by drying at 60 °C for 12 h.

### 2.3. Preparation of PANI–MoS_2_ Nanocomposite

The hierarchical PANI–MoS_2_ nanocomposites were fabricated with PANI–MoOx nanocomposites as the precursors. Subsequently, 0.40 g of PANI–MoOx nanocomposite and 0.30 g thiourea were introduced into 20 mL DI water and then transferred to a Teflon-lined autoclave at a temperature of 200 °C for 2 days. The obtained PANI–MoS_2_ sample was centrifuged and washed three times with DI water, followed by dehydration by drying at a temperature of 50 °C. As a control sample, MoS_2_ was obtained following the same method, except that (NH_4_)_6_Mo_7_O_24_•4H_2_O was used as the molybdenum source.

### 2.4. Material Characterizations

X-ray diffraction patterns with Cu Kα radiation (XRD, λ = 1.5406 Å, Rigaku D/max 2500 pc, Tokyo, Kanto, Japan), Raman spectra (Renishaw, Wotton-under-Edge, Gloucestershire, UK), and a Fourier transform infrared spectrometer (FTIR, Thermo Nicolet Nexus 670, Madison, WI, USA) were used to examine the detailed phase structure. The physical–chemical properties of the sample were studied by an X-ray photoelectron spectroscope (XPS, K-Alpha+, East Grinstead, West Sussex, UK). The surface morphologies and internal structures of the samples were visualized through a scanning electron microscopy technique (SEM, JSM-7800F, Tokyo, Kanto, Japan) with an EDS and transmission electron microscopy test (TEM, JEOL JEM-2100, Tokyo, Kanto, Japan). Thermogravimetric (TG) analysis (NETZSCH-STA409PC, Selb, Bavaria, Germany) was performed under a ramping rate of 10 °C/min from ambient temperature to 600 °C.

### 2.5. Electrochemical Measurements

The related electrochemical performance of the obtained products was exhibited via a CR2032 coin cell. The working electrode was fabricated by composition of working electrode in preparing the slurry Super P, active material, PVDF (8:1:1) by weight and then coated on carbon paper. The cell used the prepared electrode as the working electrode, zinc foil as the counter/reference electrode, and a glass fiber membrane (Whatman GF/A) as the separator. The electrolyte consisted of a 3.0 M aqueous solution of zinc trifluoromethanesulfonate (Zn(OTf)_2_). The mass of the active material on the electrodes was controlled at about 1.5 mg cm^−2^. Cyclic voltammetry (CV) and electrochemical impedance spectroscopy (EIS) measurements were carried out using an electrochemical workstation (CHI760E, CH Instruments, Inc., Austin, TX, USA) The galvanostatic charge–discharge (GCD) data and electrostatic intermittent titration technique (GITT) analyses were obtained on a Neware CT-4008 battery tester (Neware, CT-4008, Shenzhen, China) at ambient conditions (25 °C) with varying current densities.

## 3. Results

### 3.1. Structure and Morphology of PANI–MoS_2_ Composites

**Figure 1:**
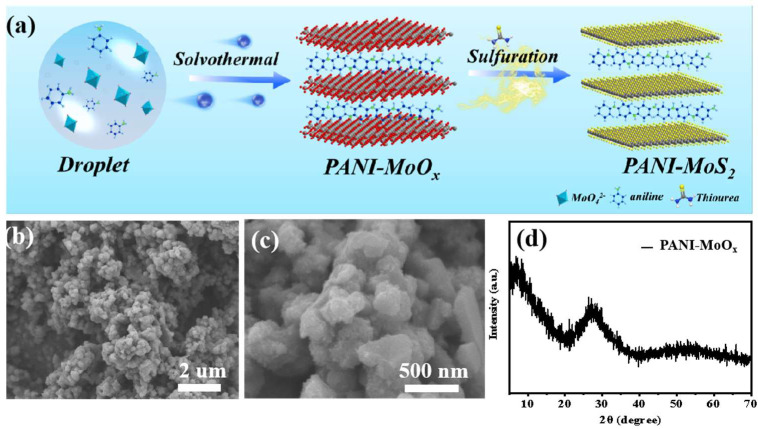
(**a**) Schematic illustration of synthesis procedure for the PANI–MoS_2_ nanocomposites; (**b**,**c**) SEM images of PANI–MoOx; (**d**) XRD pattern of PANI–MoOx.

The synthetic procedure for the PANI–MoS_2_ composites, designed as advanced electrode materials for AZIBs, is schematically illustrated in Figure 1a. Briefly, the process began with the preparation of a PANI–MoOx precursor using a hydrothermal method. Subsequently, a flower-like structure assembled with PANI–MoS_2_ nanosheets was obtained through a secondary hydrothermal reaction with thiourea as the sulfur source. The chemical reactions involved in this preparation process for the PANI–MoS_2_ composites are shown as follows:

(i) Ion exchange and coordination:
  3MoO^6^^−^ + 11H^+^ + 12HO + 14C_6_H_7_N ⇌ 7 [Mo_3_O_1__0_(C_6_H_8_N)_2_]·2H_2_O
(ii) Oxidative polymerization:
  7 [Mo_3_O_1__0_(C_6_H_8_N)_2_]·2H_2_O + (NH_4_)_2_S_2_O_8_ → PANI-MoO_x_
(iii) Vulcanization (sulfurization) reaction:
  PANI-MoO_x_ + CH_4_N_2_S → PANI-MoS_2_

The X-ray diffraction (XRD) analysis of the PANI-intercalated MoOx nanocomposites (Figure 1b) confirmed an amorphous molybdenum oxide state. The diffraction peaks (d-spacing of 1.31 nm) shifted to lower 2θ values, indicating successful intercalation of PANI into the amorphous molybdenum oxide layers [34]. The corresponding scanning electron microscopy (SEM) images at varying magnifications (Figure 1c,d) revealed inhomogeneous solid particles with dimensions below 500 nm, confirming the nanostructured morphology of the composite. Then, the flower-like structure assembled with PANI–MoS_2_ nanosheets was obtained by a further hydrothermal reaction using thiourea as the sulfur source.

**Figure 2:**
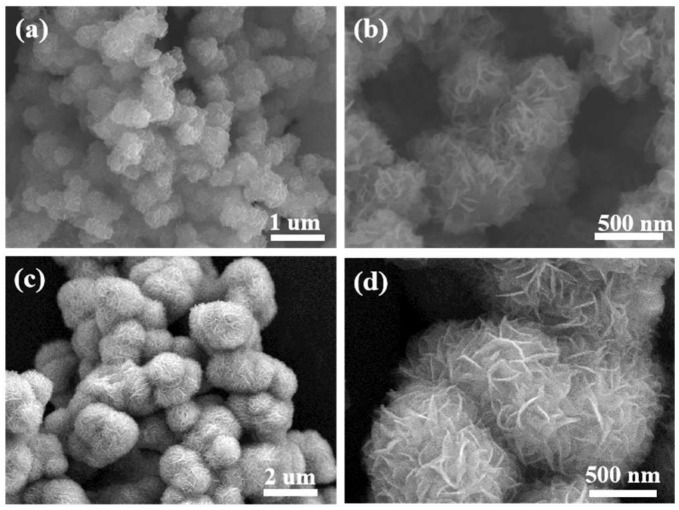
SEM images of prepared PANI–MoS_2_ (**a**,**b**) and P-MoS_2_ (**c**,**d**).

**Figure 3:**
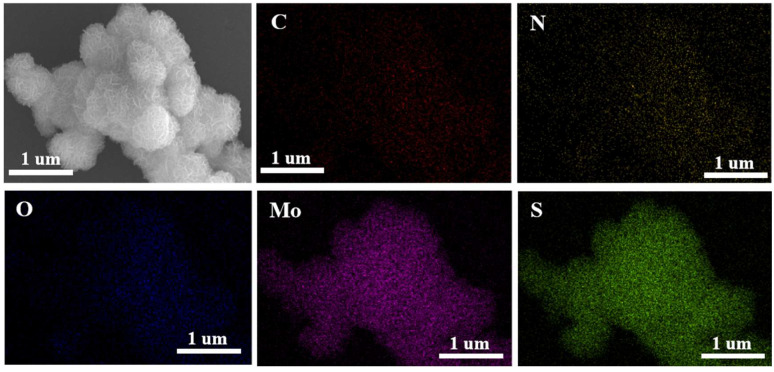
EDS mappings of PANI–MoS_2_ with C, N, O, Mo, and S elements.

The surface morphologies of PANI-intercalated MoS_2_ (PANI–MoS_2_) and pristine MoS_2_ (P-MoS_2_) composites were characterized via SEM at varying magnifications (Figure 2). The PANI–MoS_2_ composites exhibited a hierarchical nanoflower-like morphology, where spherical assemblies (radius ≈ 500 nm) were formed by interconnected nanosheets (Figure 2a,b). Notably, the PANI–MoS_2_ nanoflowers demonstrated enhanced uniformity in dispersion compared to the non-intercalated P-MoS_2_ (Figure 2c,d). The expanded interlayer spacing in the PANI–MoS_2_ architecture facilitates accelerated ion/electrolyte diffusion kinetics, thereby improving electrochemical performance [41]. Energy-dispersive X-ray spectroscopy (EDS) elemental mapping (Figure 3) confirmed the homogeneous distribution of C, N, O, Mo, and S elements within the composite. The spatially uniform distribution of C and N signals further confirmed the successful and homogeneous integration of PANI into the MoS_2_ host matrix.

**Figure 4:**
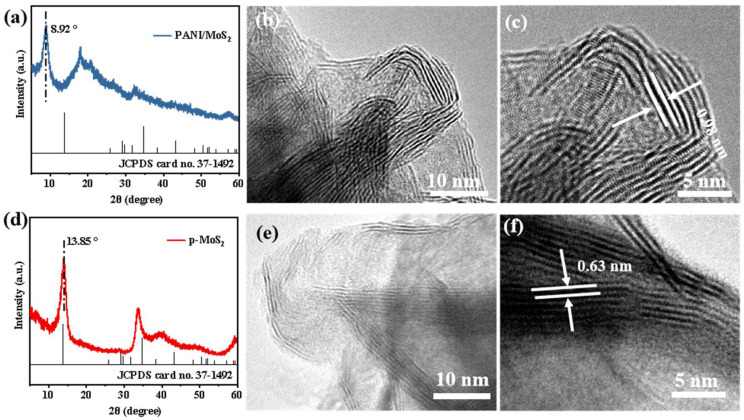
XRD patterns of PANI–MoS_2_ (**a**) and P-MoS_2_ (**d**); TEM images of PANI–MoS_2_ (**b**,**c**) and P-MoS_2_ (**e**,**f**).

Powder XRD was applied to reveal the crystallinity and crystal structure of PANI–MoS_2_ (Figure 4a) and P-MoS_2_ (Figure 4d). The pristine MoS_2_ exhibited distinct XRD diffraction peaks at 2θ = 13.85°, 33.41°, and 39.52°, corresponding to the (002), (100), and (103) crystallographic planes of the hexagonal 2H-MoS_2_ phase (space group P63/mmc, JCPDS No. 37-1492), respectively, confirming the high phase purity of the synthesized material (Figure 4a) [42]. Upon PANI intercalation, the (002) diffraction peak of the PANI–MoS_2_ sample shifted to a lower angle (from 13.85° to 8.92°) (Figure 4d), indicating a structural expansion of the interplanar distance from 0.63 nm to 0.98 nm. As expected, the high-resolution TEM (HRTEM) images confirmed this structural expansion, showing an interplanar distance of 0.98 nm from the MoS_2_ nanosheets in PANI–MoS_2_ (Figure 4b,c) compared to 0.63 nm in the P-MoS_2_ nanosheets (Figure 4e,f). These results indicate that the PANI molecules were successfully embedded into the MoS_2_ layers, forming expanded interlayer structures that overlap each other and enlarging the interlayer distance of MoS_2_. The structural expansion of PANI–MoS_2_ is expected to enhance the Zn^2+^ ions (de)intercalation and thereby optimize the charge-storage efficiency and capacity.

**Figure 5:**
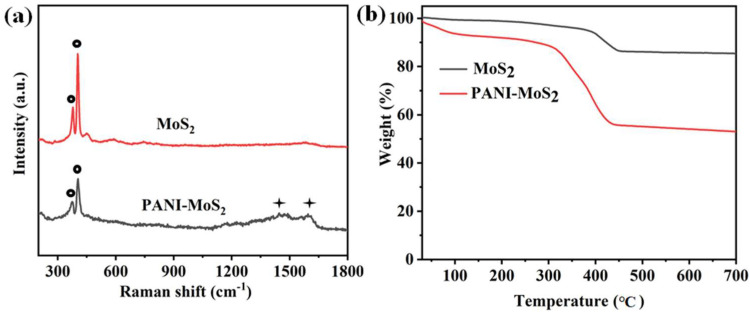
Raman patterns (**a**) and TG curves (**b**) of PANI–MoS_2_ and P-MoS_2_.

In order to further verify the intercalation of PANI into MoS_2_, Raman analyses of PANI–MoS_2_ and P-MoS_2_ were performed, respectively (Figure 5a). Characteristic peaks at 378 cm^−1^ and 404 cm^−1^ corresponded to the E12g (related to the in-plane displacement of the sulfur atoms) and A1g (related to the out-of-plane symmetric displacement of the sulfur atoms along the C-axis) modes of the MoS_2_ phase [43]. The peaks near 1450 cm^−1^ and 1590 cm^−1^ were associated with the characteristic peaks for the conducting polymer PANI in PANI–MoS_2_ as compared to pure P-MoS_2_. The vibrational characteristic peak centered at about 1450 cm^−1^ is characteristic of the C–N stretching mode of the benzene ring of PANI, while the characteristic peak at 1590 cm^−1^ is assigned to the C–C stretching vibration of PANI. To further investigate the presence of guest species PANI, a thermogravimetric analysis (TGA) was performed. The presence of PANI within the MoS_2_ interlayer was proved by the sudden mass loss caused by the thermal decomposition of PANI (Figure 5b). The weight loss between 300 and 450 °C of the TG curves was attributed to the combustion of organic molecules, indicating that PANI constituted approximately 33.5 wt% of the PANI–MoS_2_ composite, whereas the weight loss before may be ascribed to the adsorbed molecules of water on the surface and evaporation of structural water.

**Figure 6:**
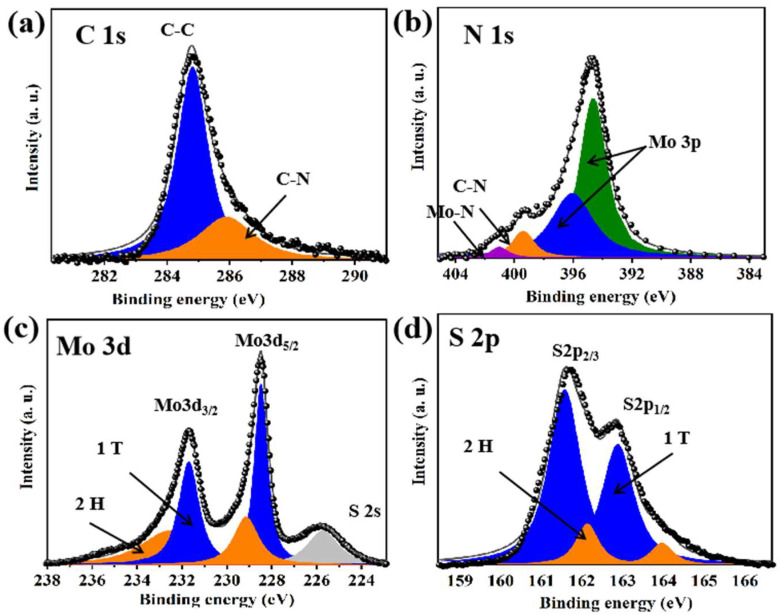
C 1s (**a**), N 1s (**b**), Mo 3d (**c**), and S 2p (**d**) high-resolution XPS spectra of PANI–MoS_2_.

The elemental and chemical composition of PANI–MoS_2_ was analyzed by X-ray photoelectron spectroscopy (XPS) (Figure 6). In Figure 6a, the high-resolution XPS spectra of C1s could be divided into two major peaks, which appeared at 282.2 eV (C-N) and 284.8 eV (C-C), respectively. The XPS N1s spectrum exhibited two characteristic peaks derived from C–N (399.5 eV) and Mo–N (401.2 eV) structures, indicating the chemical coordination between the PANI and MoS_2_ layers (Figure 6b). The Mo 3d and S 2p spectra confirmed the coexistence of 1T and 2H phases in PANI–MoS_2_, which is beneficial for maintaining structural stability during Zn^2+^ intercalation/deintercalation processes. In the high-resolution XPS spectra of elemental Mo (Figure 6c), the Mo 3d spectrum contained two related splitting peaks corresponding to Mo3d_5/2_ and Mo3d_3/2_. Notably, the deconvolution of the Mo 3d spectrum reveals two peaks: a low-binding-energy peak indicative of the 1T phase and a high-binding-energy peak associated with the 2H phase of MoS_2_, respectively [26]. Similarly, the S spectra can also be deconvoluted into contributions from the 1T and 2H phases (Figure 6d). The coexistence of these two phases in PANI–MoS_2_, as evidenced by the fitting results, contributes to maintaining the structural stability of the PANI–MoS_2_ composite during the intercalation/deintercalation process of alkali metal ions, such as Li^+^, Na^+^, and Zn^2+^. This structural configuration is beneficial for enhancing the electrochemical performance and cyclability of the electrode material.

### 3.2. Zinc Storage Properties of PANI–MoS_2_ Composites

**Figure 7:**
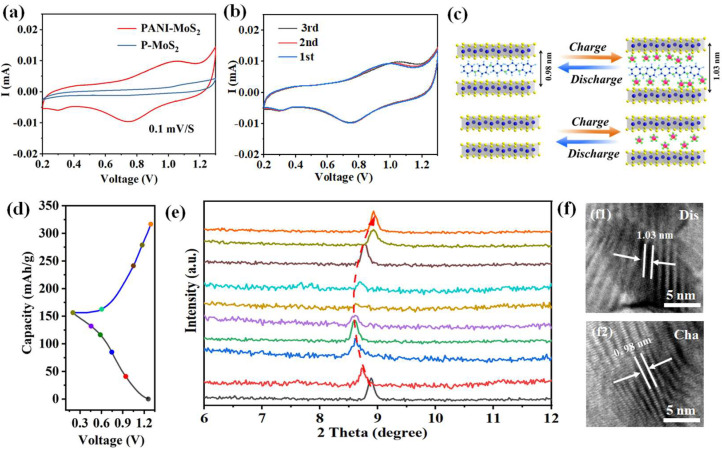
(**a**) CV curve of PANI–MoS_2_ and P-MoS_2_ at a scan rate of 0.1 mV s^−1^; (**b**) CV profiles of the PANI–MoS_2_ electrode at a scan rate of 0.1 mV s^−1^; (**c**) schematic diagram of charge and discharge of PANI–MoS_2_ and P-MoS_2_; (**d**,**e**) ex situ XRD patterns at 0.1 A g^−1^ in the discharge and charge states; (**f**) TEM images of PANI–MoS_2_ at fully discharged states (**f1**) and fully charged (**f2**).

The electrochemical performances of the PANI–MoS_2_ and P-MoS_2_ electrodes as cathodes for zinc ion storage were evaluated and compared using CR2032-type coin cells with zinc foil anodes and 3M Zn(CF_3_SO_3_)_2_ electrolytes. Initially, cyclic voltammetry (CV) tests for the PANI–MoS_2_ and P-MoS_2_ electrodes were performed at a scan rate of 0.2 mV s^−1^, as shown in Figure 7a. The CV curves showed two distinct redox reactions for PANI–MoS_2_ at ~1.1 V (oxidation of Mo⁴^+^ into Mo^6^^+^) and 0.75 V (the reduction of Mo^6^^+^ to Mo⁴^+^), which is consistent with the reversible deintercalation and intercalation of Zn^2^^+^ ions during cycling [32]. Meanwhile, pristine MoS_2_ retains a similar redox potential to PANI–MoS_2_, suggesting similar redox chemistry. Notably, PANI–MoS_2_ exhibited a significantly higher current response than P-MoS_2_, reflecting its superior Zn^2^^+^ storage capacity. Intriguingly, an additional cathodic peak at lower potentials (~0.3 V) in PANI–MoS_2_ can be attributed to the structural disorder within the Zn^2^^+^/H_2_O superlattice, facilitating the intercalation of Zn^2^^+^ originating from the PANI-induced interlayer expansion [27]. The peak is reversible across cycles (Figure 7b). The emergence of such low-potential processes underlines the key role of PANI embedding in modulating the host structure by favoring ion entry pathways and enhancing charge transfer kinetics (Figure 7c). Together, these results demonstrate that the strategic introduction of PANI can enhance the electrochemical functionality of MoS_2_ by tuning the interlayer structure and electronic environment.

To further confirm the Zn^2+^ storage mechanism of the PANI–MoS_2_ electrodes, ex situ XRD analyses during the first charge–discharge cycle were carried out at different charge and discharge states (Figure 7d,e). During the discharging process, the diffraction peaks of the PANI–MoS_2_ (002) peak became broader and weaker and shifted to a lower diffraction angle. This phenomenon indicates that Zn^2+^ was inserted into the MoS_2_ matrix to form Zn_X_MoS_2_, with a larger layer spacing than the original PANI–MoS_2_ product (0.98 nm versus 0.63 nm). During the subsequent charging process, this diffraction peak of the PANI–MoS_2_ (002) plane gradually returned to its original position, indicating that Zn^2+^ was reversibly extracted from Zn_X_MoS_2_. This indicates that PANI–MoS_2_ is highly reversible during charging and discharging. The different Zn^2+^ intercalation behaviors were further displayed by fully charged and discharged HRTEM observations in Figure 7f. The interlayer spacing for PANI–MoS_2_ showed 1.03 nm in the fully discharged state (Figure 7f1), which reverted to the original spacing after the fully charged process (Figure 7f2), corroborating the above XRD results. A similar mechanism was found in oxygen-rich defective MoS_2_ electrodes with increased layer spacing and 1T/2H phase hybridization [26].

From the above CV, non-in situ XRD and non-in situ HRTEM analyses, the possible electrochemical reactions between the PANI–MoS_2_ hybrid positive electrode and the Zn negative electrode are as follows.

Anode: XZn^2+^ + 2Xe^−^ + MoS_2_ = Zn_X_MoS_2_ (1)

Negative electrode: XZn^2+^ + 2Xe^−^ = XZn (2)

A similar mechanism is found in defect-rich MoS_2_ electrodes, oxygen-containing MoS_2_ electrodes, and MoS_2_ nanosheet electrodes with enlarged layer spacing [32,44].

**Figure 8:**
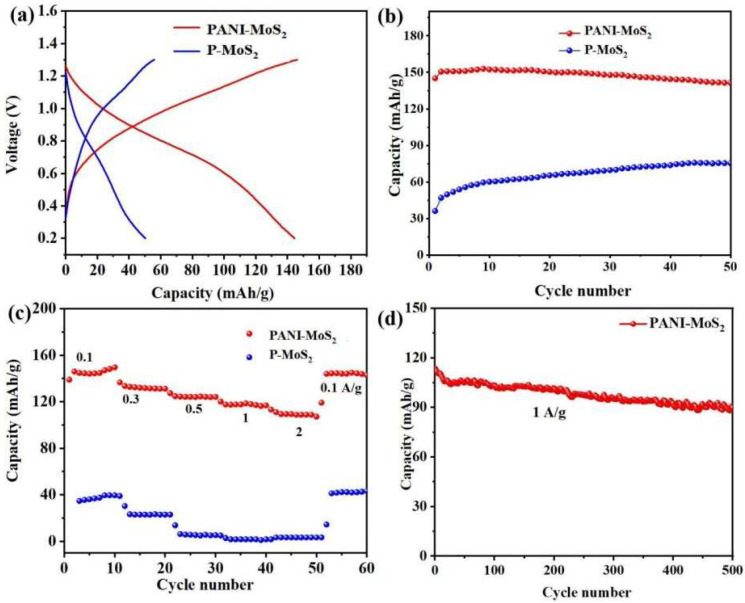
(**a**) The corresponding constant-current charge/discharge curves of PANI–MoS_2_ and P-MoS_2_ at 0.1 A g^−1^ and cycling performance at 0.1 A g^−1^ (**b**); (**c**) rate performance of PANI–MoS_2_ at different current densities; (**d**) long-term cycling performance of PANI–MoS_2_ at 1 A g^−1^.

The galvanostatic charge–discharge profiles of the PANI–MoS_2_ and P-MoS_2_ electrodes were illustrated at 0.1 A g^−1^ (Figure 8a). Clearly, the pre-intercalation of PANI markedly enhances the Zn^2^^+^ storage capability of the MoS_2_ host. The P-MoS_2_ electrode displays an exceptionally low initial discharge capacity of 37.9 mAh g^−^^1^ due to its limited layer spacing (0.63 nm), which hinders reversibility of insertion/extraction of hydrated Zn^2^^+^ ions. In contrast, the capacity of the PANI–MoS_2_ electrode was significantly improved, delivering a higher initial discharge capacity of 150.5 mAh g^−1^. The gradual increase in capacity relative to pristine MoS_2_ is undoubtedly caused by the interlayer expansion of MoS_2_. After 50 cycles, PANI–MoS_2_ still retained a capacity of 141.3 mA g^−1^, with a retention rate of 83.3% (Figure 8b). Figure 8c demonstrates the rate capability tests of the PANI–MoS_2_ and P-MoS_2_ electrodes at different current densities. The discharge storage capacities of the PANI–MoS_2_ electrode were 150.1, 115.5, 102.9, 83.9, and 58.3 mA h g^−1^ at current densities of 0.1, 0.3, 0.5, 1.0, and 2.0 A g^−1^, respectively. The specific capacity was restored to 133.9 mA h g^−1^, with 89.2% capacity retention when the current density was reset to 0.1 A g^−1^. Figure 8d shows the long-term cycling tests of PANI–MoS_2_ at a current density of 1 A g^−1^. PANI–MoS_2_ shows an initial capacity up to 113 mA h g^−1^, with capacity retention up to 81.2% of its initial storage capacity after 500 cycles. The MoS_2_ interlayer spacing expansion induced by polyaniline intercalation (≈0.98 nm) greatly exceeds the ionic radius of hydrated Zn^2+^ (0.55 nm). The special π-conjugated structure of the embedded PANI cloud shield the electrostatic interactions of the Zn^2+^ and the MoS_2_ hosts, preventing the structure from collapsing and obtaining a stable long-term cycling. Thus, the polyaniline-intercalated MoS_2_ composites exhibit superior cycling stability compared to the pristine MoS_2_, as evidenced by their attenuated capacity degradation and consistent charge/discharge voltage profiles over long cycling times. The designed interlayer spacing for the PANI–MoS_2_ electrode not only facilitates the fast diffusion kinetics of Zn^2+^ but also mitigates the repetitive structural stresses during the electrochemical process, which is essential to maintain the long-term durability of the electrodes [38].

**Figure 9:**
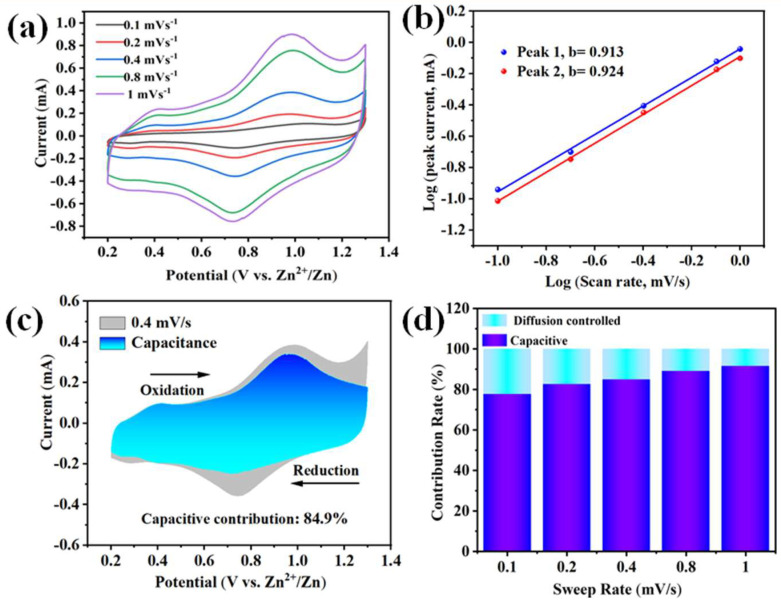
(**a**) CV curves at different scan rates of PANI–MoS_2_; (**b**) log (i) versus log (v) plots of two redox peaks of PANI–MoS_2_; (**c**) CV profile at 0.4 mV s^−1^ of PANI–MoS_2_ cathode; (**d**) capacitive contributions of PANI–MoS_2_ at different scan rates.

The electrochemical kinetics of the PANI–MoS_2_ electrode were deeply revealed by the CV curves, with different scan rates in the range of 0.1–1.0 mV s^−1^ (Figure 9a). Obviously, the peak potential shift of the PANI–MoS_2_ composites during Zn^2^^+^ insertion/de-insertion is small, even at higher scan rates. This observation demonstrates the superior electrochemical kinetics and enhanced reversibility for PANI–MoS_2_ electrodes, as evidenced by the negligible polarization effects. The relationship between tested peak current (i) and the scanning rate(v) was revealed by the formula i = av^b^, where a and b indicate adjustable parameters. A value of b of 1 indicates surface capacitive behavior, while b = 0.5 indicates a completely diffusion-controlled behavior. By calculating the log(i) versus log(v) curves, the b values of 0.913 and 0.924 for the redox peaks suggested a dominant pseudocapacitive contribution (Figure 9b). The high b value of the PANI–MoS_2_ electrode during both the discharge and charge processes suggests a synergistic contribution of pseudocapacitive surface redox reactions and ion insertion/extraction processes in governing dominant charge storage mechanisms. The ratio of diffusion-limited and pseudocapacitive contributions of the Zn^2+^ ion storage mechanisms can be further resolved by the formula i = k_1_v + k_2_v^1/2^ and i/v^1/2^ = k_1_v^1/2^ + k_2_, where i and v represent the current (A g^−1^) and scan rate (mV s^−1^), respectively. Notably, ≈84.9% current arises predominantly from pseudocapacitive processes at 1.0 mV s^−1^ (Figure 9c). The proportions of capacitive (k_1_v) and diffusion-controlled (k_2_v) contributions can be approximated by a gradual increase in the proportion of capacitance from 76.5% to 89.6% as the scan rate is increased from 0.1 mV s^−1^ to 1.0 mV s^−1^ (Figure 9d). The results indicate that the reaction process of PANI–MoS_2_ at a high scan rate is mainly related to the surface capacitance behavior, and this higher capacitance storage is conducive to the improvement of the multiplicity performance and cycling stability for AZIBs.

**Figure 10:**
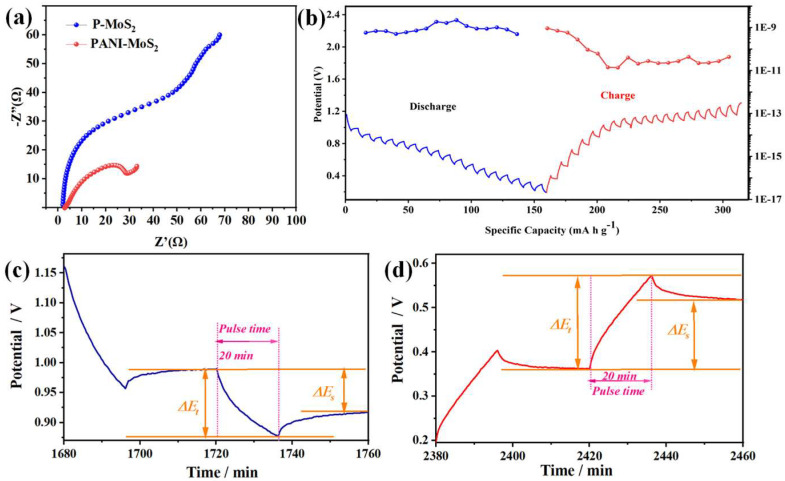
(**a**) Nyquist plots of P-MoS_2_ and PANI–MoS_2_ electrodes at 5 cycles; (**b**) GITT plot at 0.5 A g^−1^ and the Zn^2+^ diffusion coefficient of PANI–MoS_2_; (**c**,**d**) close-up view of the GITT curve for PANI–MoS_2_.

The fast Zn^2+^ diffusion kinetics in PANI–MoS_2_ are also explored by electrochemical impedance spectra (EIS) and constant-current intermittent titration (GITT). The fitted EIS data in Figure 10 reveals that the PANI–MoS_2_ electrode exhibits a significantly lower Rct value (29.4 Ω) compared to the P-MoS_2_ electrode (Rct = 51.2 Ω). This reduction indicates the beneficial role of PANI intercalation in enhancing the charge transfer kinetics at the electrode–electrolyte interface of AZIBs (Figure 10a). Figure 10b–d show the results of the diffusion coefficients of zinc ion (DZn^2+^) for PANI–MoS_2_ electrodes at a pulse time of 20 min, relaxation time of 30 min, and pulse current of 100 mA g^−1^. Notably, the average DZn^2+^ values for PANI–MoS2 electrodes are calculated as 1.36 × 10^−9^–9.2 × 10^−10^ cm^2^s^−1^ (discharge) and 4.9 × 10^−10^–2.2 × 10^−9^ cm^2^s^−1^ (charge), respectively. Ion-transport studies at the electrodes of AIBs based on conducting polymers show a similar order of magnitude (10^−9^–10^−12^ cm^2^ s^−1^), which is in agreement with our experimental systems [27,38]. The significantly high DZn^2+^ values emphasize the key role of interlayer expansion and increased electronic conductivity via PANI intercalation in facilitating Zn^2+^ intercalation kinetics, thus optimizing the electrochemical performance for Zn^2+^ storage.

## 4. Conclusions

In summary, the strategic intercalation of conductive polyaniline (PANI) into MoS_2_ interlayers successfully yielded PANI–MoS_2_ composites with a significantly expanded interlayer spacing of 0.98 nm. Systematic structural characterization confirmed that PANI intercalation induced interlayer expansion, enhanced conductivity, and introduced abundant redox active sites, thereby enhancing Zn^2^^+^ diffusion kinetics. Caused by these structural and electronic modifications, the PANI–MoS_2_ cathode delivered exceptional electrochemical performance, including a high reversible capacity of 150.1 mA h g^−1^ at 0.1 A g^−1^ and remarkable rate capability (113.3 mA h g^−1^ at 1 A g^−1^). The electrode also demonstrates outstanding cyclability, retaining 81.2% of its capacity after 500 cycles at 1 A g^−^^1^. Ex situ XPS and XRD analyses suggested the highly reversible Zn^2^^+^ intercalation/extraction within the PANI–MoS_2_ host structure. This work establishes a novel paradigm for engineering cathode architectures with optimized ion-transport pathways and electronic conductivity, advancing high-performance aqueous ZIBs for scalable energy storage.

## Data Availability

As these data are also part of an ongoing study, it is not currently possible to share the raw/processed data required to replicate the results of these studies.
